# Allelic Survey and Population Genomics in a Hotspot of Diflubenzuron Resistance in the Mosquito *Culex pipiens*

**DOI:** 10.3390/insects17080877

**Published:** 2026-08-21

**Authors:** Flavio Piras, Valentina Lucchesi, Arianna Puggioli, Romeo Bellini, Daniele Porretta, Valentina Mastrantonio

**Affiliations:** 1 Department of Environmental Biology, Sapienza University, 00185 Rome, Italy; flavio.piras@uniroma1.it (F.P.); valentina.lucchesi@uniroma1.it (V.L.); daniele.porretta@uniroma1.it (D.P.); 2 Medical and Veterinary Entomology, Centro Agricoltura Ambiente “G. Nicoli”, 40014 Crevalcore, Italy; apuggioli@caa.it (A.P.); rbellini@caa.it (R.B.)

**Keywords:** chitin synthesis inhibitors, ddRAD, gene flow, target-site resistance, mosquitoes, population genomics

## Abstract

The long-term efficacy of vector control programs is seriously hampered by the evolution of insecticide resistance. In this scenario, assessing the distribution, frequency, and drivers of resistance spread is crucial to planning effective control programs. Here, we addressed these issues in the mosquito *Culex pipiens*, focusing on the Emilia-Romagna region (Italy), a known hotspot of diflubenzuron (DFB) resistance. First, *Cx. pipiens* larvae were screened for point mutations conferring DFB resistance to capture the distribution and frequency of resistance-associated alleles across the region. To this end, a fragment of the chitin synthase 1 gene spanning the mutated site was sequenced. Then, by using population genomics, we investigated the vector population structure in this area. Our findings revealed that resistance-associated alleles maintain a focal geographic distribution in the region, with higher mutation frequencies in the central-eastern than in the western provinces. Also, we observed that the populations of *Cx. pipiens* are a highly homogeneous gene pool and can be interconnected across the region, highlighting the risk of resistance alleles spread across Emilia-Romagna. Overall, this study stresses the importance of systematically surveying resistance-associated alleles and their integration with population genomic data to gain relevant information for resistance management.

## Simple Summary

The mosquito *Culex pipiens* is a primary disease vector worldwide, and chemical insecticides have been the most widely employed control tool. Recently, insecticide resistance to diflubenzuron, a larvicide commonly used in mosquito control, has been documented in *Cx. pipiens* populations from the Emilia-Romagna region, Italy. In this study, we furnished an updated picture of the distribution and frequency of resistance-associated alleles and, by exploiting population genomics, we investigated the population structure across the region. Our results showed that diflubenzuron-resistance-associated alleles are still present in Emilia-Romagna and that their relative frequency is higher in central-eastern than in western provinces. Furthermore, they revealed a highly homogeneous gene pool across the region, posing the risk of the spread of resistance alleles. Overall, this study highlights the importance of continuously monitoring *Cx. pipiens* populations and, from an applied perspective, it provides relevant information to plan effective control programs.

## 1. Introduction

The evolution of insecticide resistance is a global threat challenging the control of harmful arthropod species. The number of insecticide-resistant species has rapidly increased over the years, and today, more than 600 species resistant to at least one insecticide have been reported, raising concern about the efficacy of multiple insecticide classes [1,2].

Insect growth regulators (IGRs) are a chemically heterogeneous group that includes microbial-derived pyrimidine-nucleoside peptides, benzoylureas (BPUs), oxazolines, and thiadiazines [3]. All these compounds interfere with chitin biosynthesis, leading to cuticle malformations, egg-hatching defects, and abortive molting [3]. These molecules are considered to have no or only mild effects on non-target species and therefore have been widely used as an alternative tool in agricultural and public health control programs [4]. However, evidence of IGR resistance has recently been reported in some species, such as *Tetranychus urticae*, *Plutella xylostella*, and *Cydia pomonella* [5,6,7]. Molecular analyses revealed that non-synonymous point mutations in the chitin synthase 1 gene (chs-1) are the key mechanism underlying these resistant phenotypes [5,6,7]. Recently, the same mutations were detected in populations of *Culex pipiens*, documenting the first record of this resistance in mosquitoes [8].

*Cx. pipiens* is the primary mosquito vector in the Mediterranean region, as it transmits several zoonotic diseases, such as West Nile fever and canine lymphatic filariases [9,10]. In 2015, resistant individuals to the IGR diflubenzuron (DFB) were first reported in Italian populations of *Cx. pipiens* from the Emilia-Romagna region (Italy) [8]. Genetic analyses revealed that while the susceptible individuals carry a gene sequence encoding for the isoleucine at position 1043 of the chs-1 gene (I1043 hereafter), the resistant individuals carry gene sequences encoding for the amino acids leucine (I1043L), methionine (I1043M), and/or phenylalanine (I1043F). Functional validation by genome editing and insecticide bioassays revealed that these three mutations confer a striking level of resistance to DFB, with resistance ratios ranging from 20- to >15,000-fold [8,11,12]. After their detection in Emilia-Romagna, DFB-resistant mutations have been reported in other regions of Northern Italy [13] and in several other Mediterranean countries, including France, Turkey, Crete, and Cyprus, highlighting a broader distribution of DFB resistance [14,15,16,17].

Notably, the Emilia-Romagna region remains a major hotspot of resistance within this distribution. In this area, all the three mutations linked to DFB resistance have been detected, and analyses of allele frequencies indicate that resistant individuals may account for more than 80% in some provinces [14,18]. Interestingly, a highly focal distribution of DFB resistance has been depicted across the region, with the eastern provinces exhibiting higher frequencies of resistant alleles than the western ones [14,18]. This pattern highlights two major priorities: the continuous monitoring of *Cx. pipiens* populations to provide a real-time picture of DFB-resistance-associated alleles and the assessment of the risk of further resistance diffusion to protect susceptible populations. Regarding the latter, estimating population connectivity is crucial because of the well-known role of gene flow as a primary driver of allele spread [19].

In this paper, we aim to address both these issues. Since 2019 [18], no studies have comprehensively addressed the distribution of DFB-resistance alleles in *Cx. pipiens* populations across the Emilia-Romagna region, and most importantly, to our knowledge, no data has ever been collected on the genetic population structure of this species using nuclear markers within the region. To fill these gaps, we used Sanger DNA sequencing of the chs-1 gene to screen *Cx. pipiens* populations across Emilia-Romagna for DFB-resistance alleles. Then, we used a population genomic approach to investigate the vector population structure in this area. Genomic approaches have revolutionized our ability to study population genetics in insects, allowing us to analyze not just single or a few markers but thousands of loci spread across the genome [20,21]. Population genomics studies are now essential for assessing genetic profiles, gene flow, and the spread of resistance traits [22,23]. These approaches have already enabled the characterization of the population structure and dynamics of agricultural pests and disease vectors [24,25,26,27], offering a valuable contribution to improving management and control programs for species of economic and medical importance [28,29]. Here, we exploited these potentialities to assess the level of connectivity between *Cx. pipiens* populations across the region.

## 2. Materials and Methods

### 2.1. Mosquitoes

**Figure 1:**
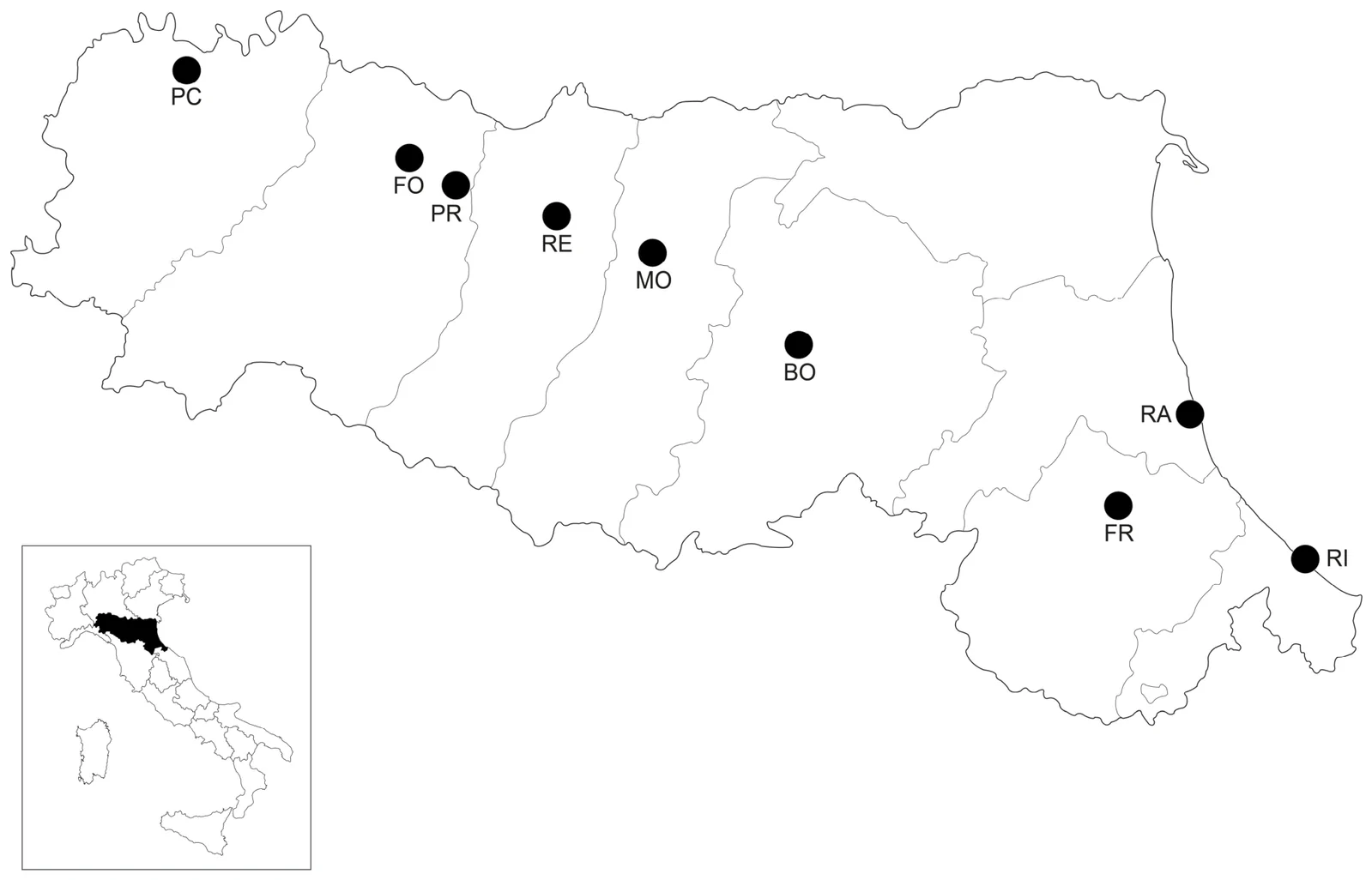
**Map of the localities sampled across the Emilia-Romagna region**. PC: Piacenza; FO: Fontevivo; PR: Parma; RE: Reggio-Emilia; MO: Modena; BO: Bologna; RA: Ravenna; FR: Forlì; RI: Rimini.

*Culex pipiens* fourth-stage larvae were collected during the summer of 2023–2024 from eastern and western provinces of the Emilia-Romagna region (Northern Italy) (Table 1, Figure 1). In each locality, multiple breeding sites were sampled. The larvae were identified using morphological keys [30] and stored in 90% ethanol for subsequent genetic analyses.

### 2.2. Analysis of the Chitin Synthase 1 Gene

Total genomic DNA was extracted from single *Culex pipiens* larvae using the C-TAB protocol as described in Doyle and Doyle [31]. Partial sequences of the chs-1 gene were obtained through amplification by Polymerase Chain Reaction (PCR). The primer pair CHSseqF (5′-CCGCGTTCAAGATTGACAACTGG-3′) and CHSseqR (5′TCCAGTAGGGGTTCGTCAGG-3′) was used to amplify a product of 825 base pairs (bp) spanning site 1043. The PCR reaction was performed in a volume of 25 μL containing 5 ng DNA, 2.5 μL of 10× buffer, 0.4 mM dNTPs, 0.4 μM primers and 2U of the DreamTaq Green DNA Polymerase (Thermo Scientific, Waltham, MA, USA). The PCR cycling procedure was 95 °C for 5 min, 30 cycles of 94 °C for 30 s, 60 °C for 30 s, and 72 °C for 1 min, and a final extension of 72 °C for 10 min. PCR products were run on a 1% agarose gel by electrophoresis to verify successful amplification. Amplified samples were sent to an external service for sequencing (Microsynth AG, Balgach, Switzerland) using the primer CP_kkv_R1 (5′-ACGTTTGCGGGTGTGATGTC-3′). All individuals analyzed were double sequenced to check for consistency. Sequences were edited and aligned using Chromas 2.6.6 (Technelysium, Helensvale, Australia) and ClustalX 2.0 [32], respectively.

### 2.3. ddRAD Sequencing, Mapping and SNP Calling

To analyze the genetic structure of *Culex pipiens* populations across the Emilia-Romagna region, a ddRAD (double digest restriction-site associated DNA) technique was employed. Genomic libraries from 74 mosquitoes were prepared at IGAtech (Udine, Italy) following a modified version of the protocol by Peterson et al. [33]. Briefly, 125 ng of DNA underwent double enzymatic digestion with *SphI* and *MboI* enzymes. Then, the sequencing was performed on an Illumina NovaSeq 6000 platform (Illumina, San Diego, CA, USA) using a 135-bp paired-end configuration (150 cycles). After demultiplexing, the quality of the raw data was assessed using FastQC and MultiQC software [34,35]. Filtering was performed using the process_radtags module of Stacks v2.68 [36,37], eliminating low-quality reads or those lacking the expected restriction site.

The filtered sequences were mapped to the *Cx. pipiens* reference genome idCulPipi1.15 [38] using the BWA-MEM v0.7.19 algorithm [39], while SAMtools v1.22.1 [40] was used for alignment management and the calculation of the mapping related statistics. Following alignment, the Stacks gstacks module [37] was used for loci assembling, as well as for SNP and individual genotype calling, using only aligned reads with a minimum mapping quality of 20 (--min-mapq 20).

### 2.4. SNP Filtering

The gstacks output was processed through the Stacks populations module [37] to retain high-confidence markers for the subsequent population genetic analyses. To this end, we filtered individual SNPs with a minimum read depth of 5×, present in at least 70% of individuals, and with a minor allele frequency < 0.05 (--min-maf 0.05) to exclude rare alleles or potential sequencing artifacts, ensuring the dataset captured shared, common neutral variation. Moreover, we discarded loci with observed heterozygosity greater than 0.7 to avoid including multi-copy sequences or paralogs. Finally, we kept the first single SNP per locus to ensure reproducibility of the dataset using the --write-single-snp parameter and used the --smooth parameter to calculate population genomics indexes such as nucleotide diversity (π). PLINK v1.90b7.7 [41] was used for the last filtering phase of our dataset. First, we checked for the presence of related individuals by setting the --rel-cutoff parameter to 0.125, and then we pruned SNPs in linkage disequilibrium using the --indep-parwise parameter set to 50 10 0.2. To avoid technical bioinformatic artifacts, such as false genetic subdivision or artificial clustering in PCA and ADMIXTURE analyses driven by uneven data patterns, we implemented stringent missingness thresholds as a best practice. In detail, we removed individuals with more than 25% missing data, and after having removed all the loci affected by more than 5% missing data, we obtained our final unbiased and reliable Variant Calling Format (VCF) file used for all the downstream population genetic analyses of this study.

### 2.5. Population Genetic Analyses

To evaluate the population genetic structure and within-population diversity, we calculated Wright’s fixation indices (F_ST_ and F_IS_) and checked for deviations from the Hardy–Weinberg Equilibrium (HWE). The hierfstat R package [42] was used to calculate the inbreeding coefficient (F_IS_), the F_ST,_ and the deviations from HWE for each locus within each population, using a permutation-based approach (1000 replicates) via the pegas R package [43]. To account for multiple testing, *p*-values were adjusted using the False Discovery Rate (FDR) method.

Then, to evaluate whether an isolation-by-distance (IBD) scenario was supported by our dataset, we tested IBD at the population levels. Pairwise genetic distances (F_ST_/(1 − F_ST_)) were calculated in hierfstat [42], with negative values set to zero and correlated against the geographic distances (calculated in km from the geographical coordinates of the samples) using a Mantel test (9999 permutations) with the vegan R package [44].

The population genetic structure and clustering were assessed using both Discriminant Analysis of Principal Components (DAPC) [45] and ADMIXTURE [46], aiming to identify the most reliable number of clusters (K). The DAPC was calculated by the adegenet v2.1.11 R package [47], whereas ADMIXTURE v1.3.0 was run with 20 independent replicates for each K (ranging from 1 to 8), using the --cv parameter to calculate the cross-validation error (CV) across the multiple runs. ADMIXTURE results were submitted to Pong v1.5 software [48] for an additional evaluation of the reliability of each run per K chosen and better graphic visualization.

### 2.6. Genomic Scan to Identify Outliers Associated with DFB Resistance

To consider the potential effect of the chs-1 locus on the genetic differentiation of the populations under analysis, we first determined the exact physical position of the target locus by aligning its nucleotide sequence to the *Cx. pipiens* reference genome idCulPipi1.15 [38] using the BLAST alignment tool v2.17.0 [49].

Then, to explore the architecture of genetic differentiation at the chromosomal scale among the six populations, the F_ST_ was calculated employing a 20 kb sliding window approach with a 5 kb step size via VCFtools v0.1.17 [50]. This parameterization was specifically chosen to optimize the signal-to-noise ratio. The graphical modeling of the genomic profiles through local regression (LOESS, span = 0.1) was conducted in the R environment using the tidyverse and data.table packages [51].

To test whether chs-1 signatures deviated from neutral background variation, their multiallelic genotypes were converted into a collapsed biallelic model by grouping all resistance variants (M, L, and F) into a single resistant allele (R) against the wild-type susceptible allele (S). This functional approach aligns the data structure with the ddRAD-seq dataset and prevents mathematical distortions typical of highly heterozygous loci [52]. Differentiation was quantified using two sample-size-weighted metrics: global F_ST_ across all populations and local allele frequency contrast (Δ*p*), defined as the absolute frequency difference between a target population and the mean of the remaining five. Finally, empirical *p*-values were calculated as the proportion of background ddRAD SNPs showing differentiation equal to or greater than chs-1. Loci falling within the ≥95th empirical percentile (p≤0.05) were identified as significant outliers probably under local selection, with loci exceeding the 99th percentile (p≤0.01) classified as strong outliers.

## 3. Results

### 3.1. Distribution and Frequency of Alleles Associated with DFB Resistance

**Figure 2:**
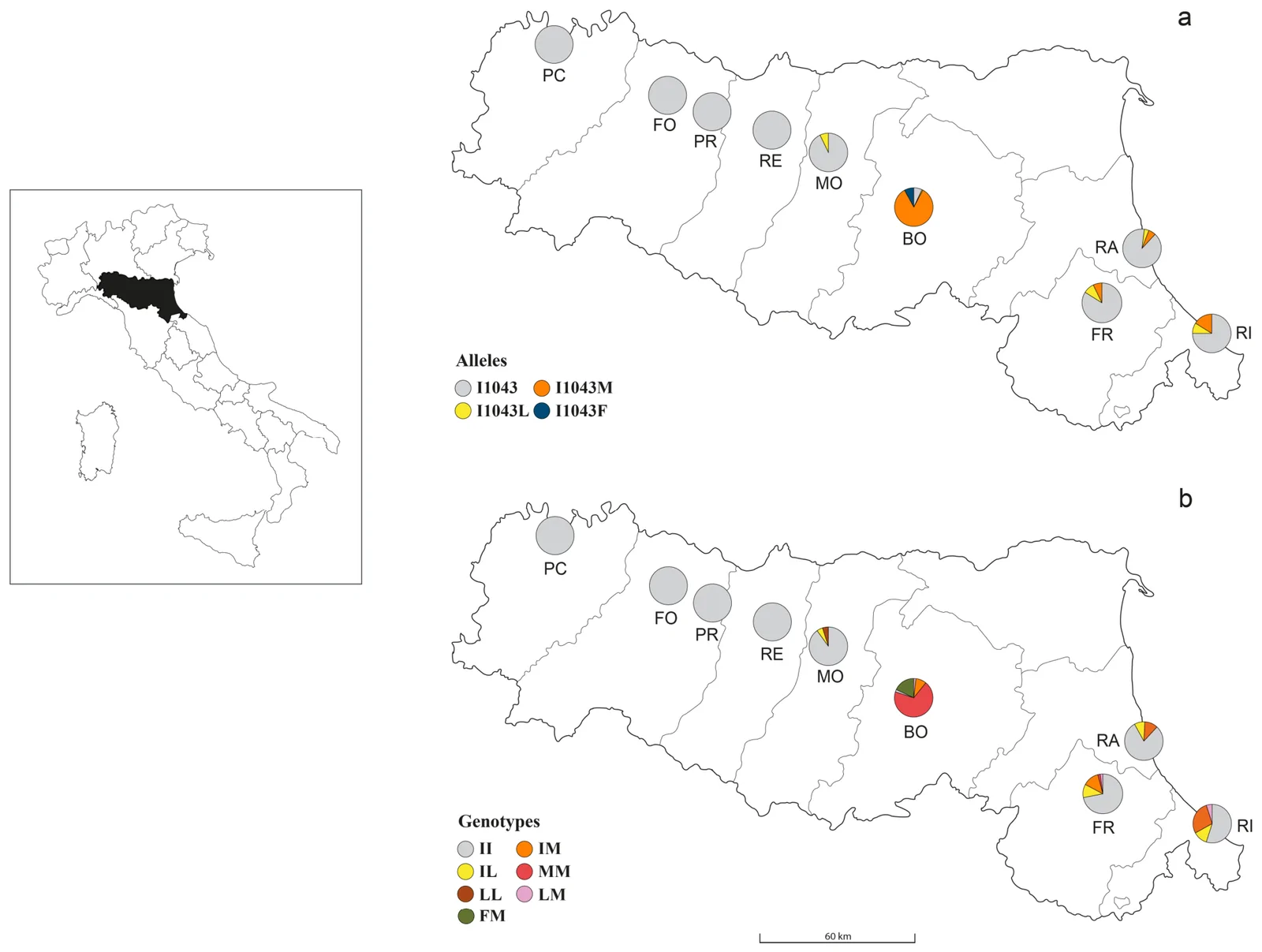
**Distribution and frequency of DFB-resistance-associated alleles in the Emilia-Romagna region.** (**a**) Allele relative frequencies observed in the sampled *Cx. pipiens* populations. (**b**) Genotype relative frequencies observed in the sampled *Cx. pipiens* populations. II = homozygous genotype for the I1043 susceptible allele; IL = heterozygote genotype I1043/I1043L; LL = homozygous genotype I1043L/I1043L; IM = heterozygote genotype I1043/I1043M; MM = homozygous genotype I1043M/I1043M; LM = heterozygote genotype I1043L/I1043M; FM = heterozygote genotype I1043F/I1043M. Localities are coded as in Figure 1.

A total of 307 *Cx. pipiens* mosquitoes were analyzed for the detection of DFB-resistant alleles (Table 1, Tables S1 and S2). DFB mutations were detected in five of the nine populations analyzed. Specifically, the I1043L mutation was detected in five populations, with relative frequencies ranging from 0.01 to 0.09 (Figure 2a, Table S1). The I1043M mutation was found in four populations, with relative frequencies ranging from 0.06 to 0.84, while the I1043F mutation was found exclusively in the Bologna locality, where it reached a frequency of 0.08 (Figure 2a; Table S1). The I1043M and I1043L mutations were found in homozygous and heterozygous genotypes, associated with both susceptible and resistant alleles, while the I1043F allele was found only in heterozygous genotypes (Figure 2b; Table S1).

### 3.2. ddRAD Sequencing

A total of 74 *Cx. pipiens* individuals were sequenced, producing 212 million reads with an average of 2.8 million reads per individual. Initial locus assembly and variant discovery yielded a total of 842,382 loci aligned to the reference genome. After applying Stack population filters, 816,366 loci were discarded, leaving a subset of 26,016 retained loci, with a mean genotyped length of 194.99 bp (SE ± 0.43 bp) each. Following site-level filtering and the exclusion of genotypes that fell below the 5× minimum threshold, 12,012 SNPs were retained. Subsequently, 2818 SNPs were pruned by PLINK because of linkage disequilibrium, and 7 individuals were removed due to more than 25% missing data, as well as 5545 SNPs due to missing genotype data. No individuals were found to be related, so the final dataset obtained after applying the Stacks population module and PLINK filtering phase accounted for 67 individuals (the 7 individuals removed included 3 individuals from Modena, 1 individual from Bologna (2024), 2 individuals from Forlì (2023) and 1 individual from Rimini) and 3556 variant sites.

### 3.3. Genetic Diversity and Population Genetic Structure

Intra-population genetic diversity was calculated by Stacks using both variant and fixed nucleotide positions. Genome-wide nucleotide diversity (π, pi_all) was low in each sampled population, ranging from 6 × 10^−4^ (Modena, Bologna, Forlì, Ravenna, Rimini) to 6.1 × 10^−4^ (Fontevivo).

**Figure 3:**
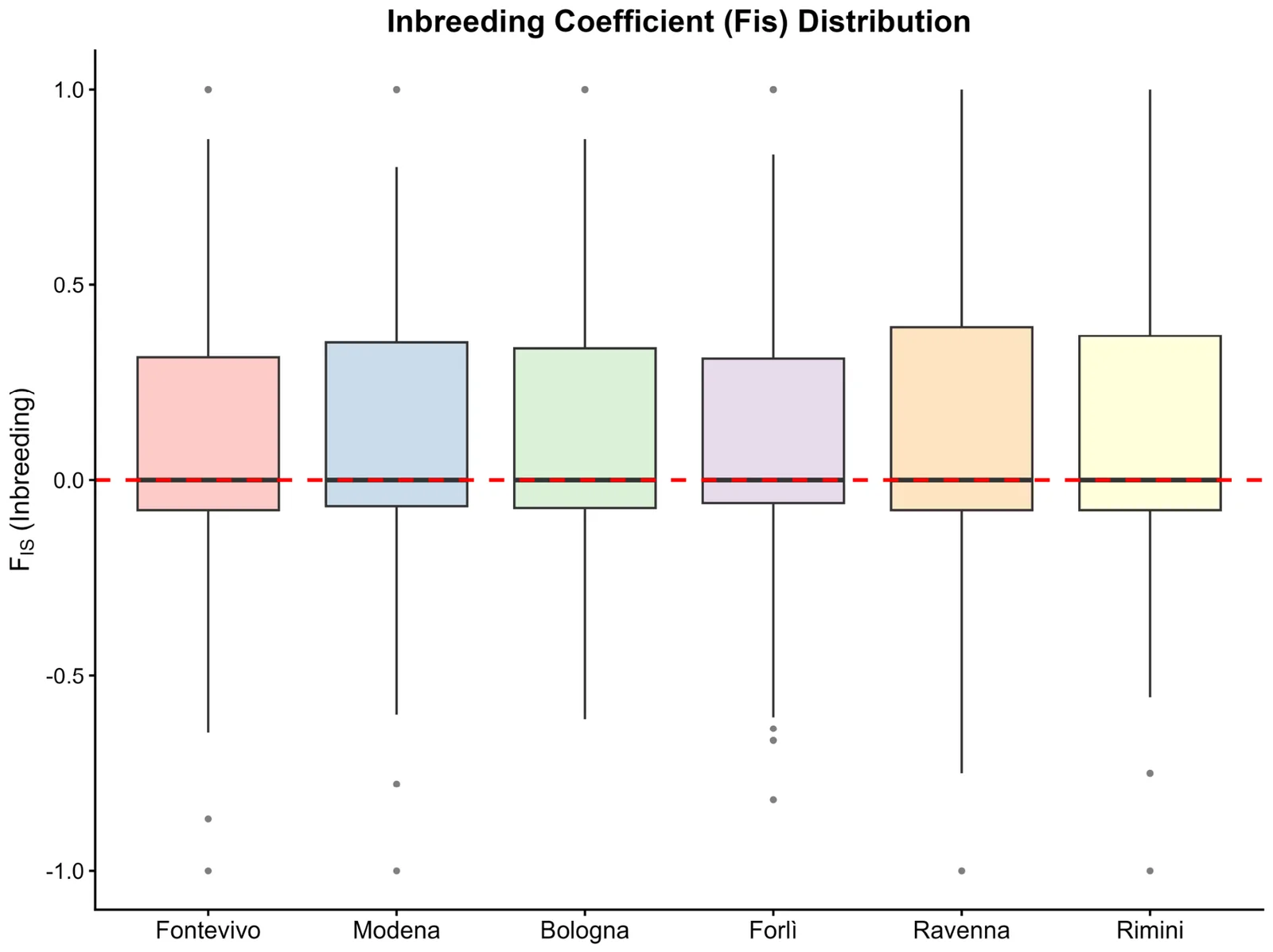
**Population-level F_IS_ distribution**. Boxplots show the distribution of F_IS_ values for each analyzed population (reported on the *x*-axis). The dashed red line shows zero, the central black lines inside the boxes denote the medians, boxes bound the interquartile ranges, and dots indicate outliers.

The inbreeding coefficient (F_IS_) analysis showed that all populations had median values near zero. Although some loci exhibited high F_IS_ values, the overall distribution remained symmetric around the equilibrium line (Figure 3). The pattern of genetic stability of the populations was further confirmed by the HWE test, which, after FDR correction, showed that the percentage of loci deviating significantly from the HWE was low across all sites. Specifically, Modena, Ravenna, and Rimini showed no loci in disequilibrium, while Fontevivo, Bologna and Forlì exhibited minor deviations corresponding to 3.31%, 2.51% and 4.12%, respectively.

**Figure 4:**
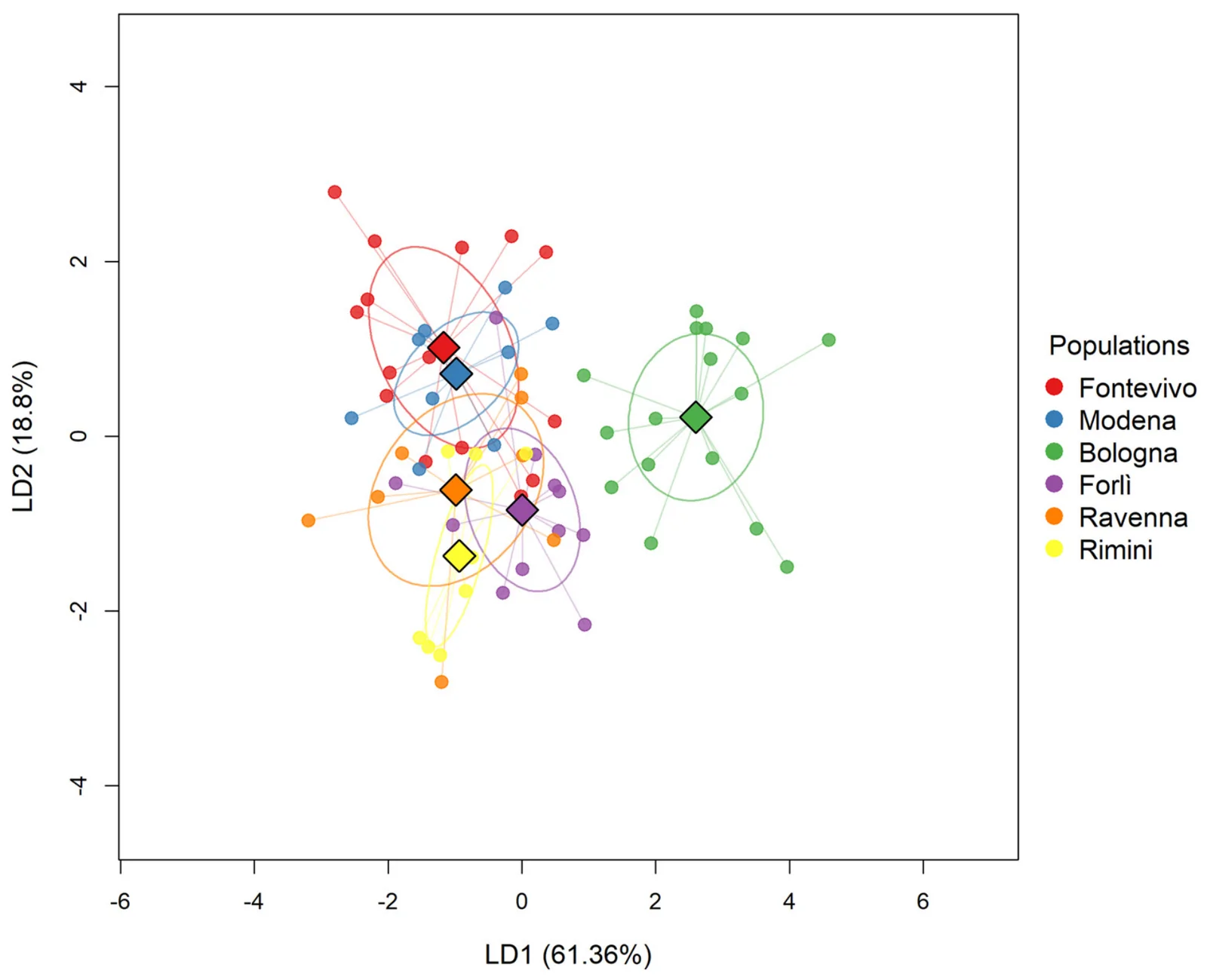
**Population clustering via Discriminant Analysis of Principal Components (DAPC).** The scatter plot illustrates the genetic relationships among individuals based on the first two discriminant functions (LD1 and LD2), which account for 61.36% and 18.8% of the total genetic variance, respectively. Dots represent individual genotypes, ellipses define the 95% confidence intervals for each population cluster, and colored diamonds represent group centroids. The color-legend for the populations analyzed is on the right.

**Figure 5:**
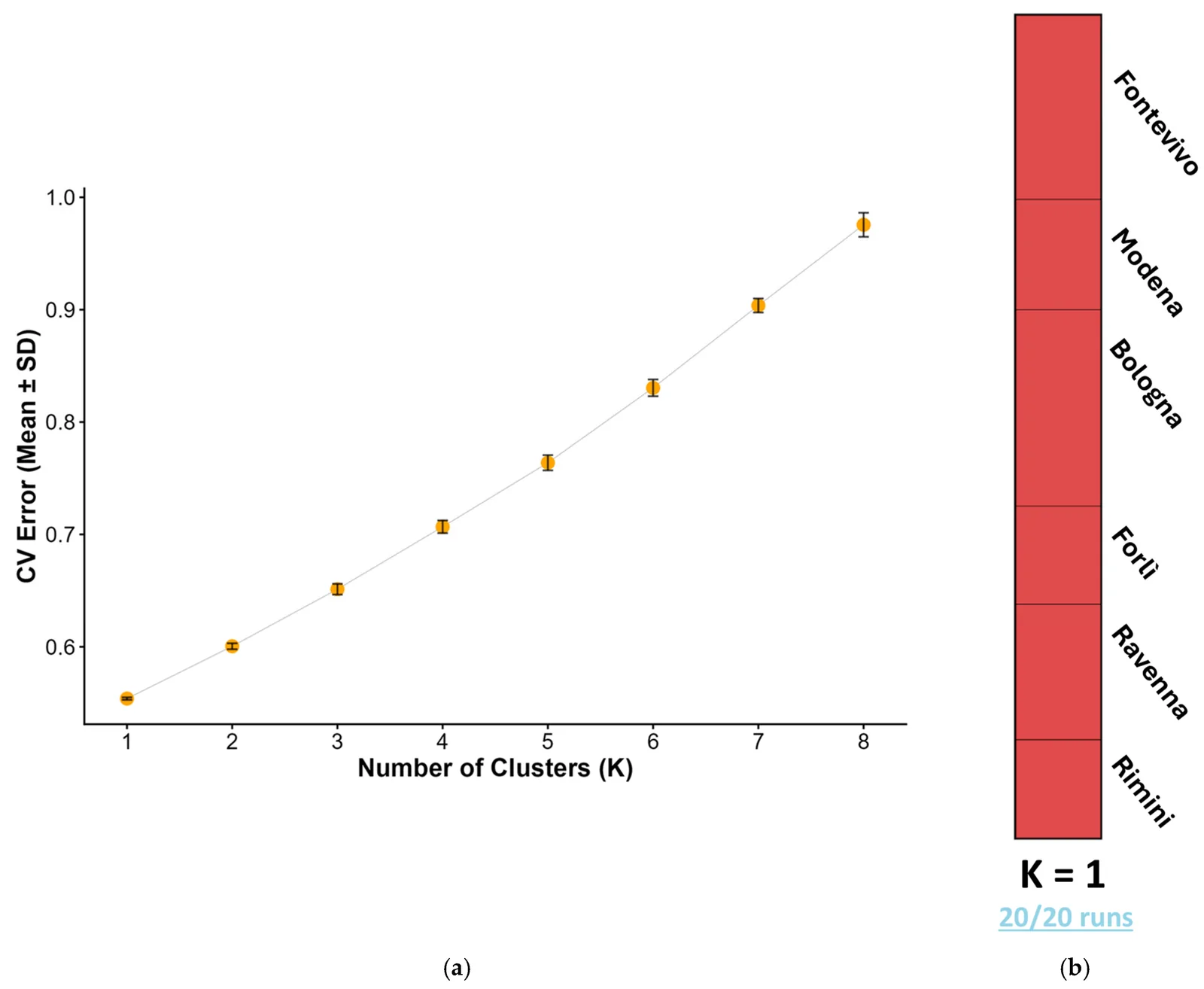
Cross-validation error and ADMIXTURE plot. (**a**) CV error plot showing the best K value; (**b**) Pong output showing the structure of the six target populations analyzed, reported at the bottom, by the best K. Individual coancestry coefficients are plotted based on a consensus of 20/20 successful iterations.

With respect to the overall population structure, the DAPC analysis, consistent with the ADMIXTURE analysis, indicated that the best clustering option is a single group, that is, K = 1 (Figure 4 and Figure 5). In the case of the admixture analysis, this finding was further confirmed by the lower cross-validation error (Figure 5a) and by the 20 consistent replicates out of the 20 calculated by the Pong software v1.5(Figure 5b), unlike higher values of K, which are characterized by high statistical uncertainty (Figure S1). However, the Bologna group showed a slight separation from the large homogeneous regional cluster along the first discriminant axis (LD1 61.36%), whereas on the second discriminant axis, a subtle north–south separation between the clusters was detected (LD2 18.8%) (Figure 4). To check for the potential effects of the filtering options, the same population structure analyses were repeated on the initial VCF file containing 12,012 SNPs from the population module. The results remained identical, consistently converging on a single group as the best clustering option (Figures S2–S4).

**Figure 6:**
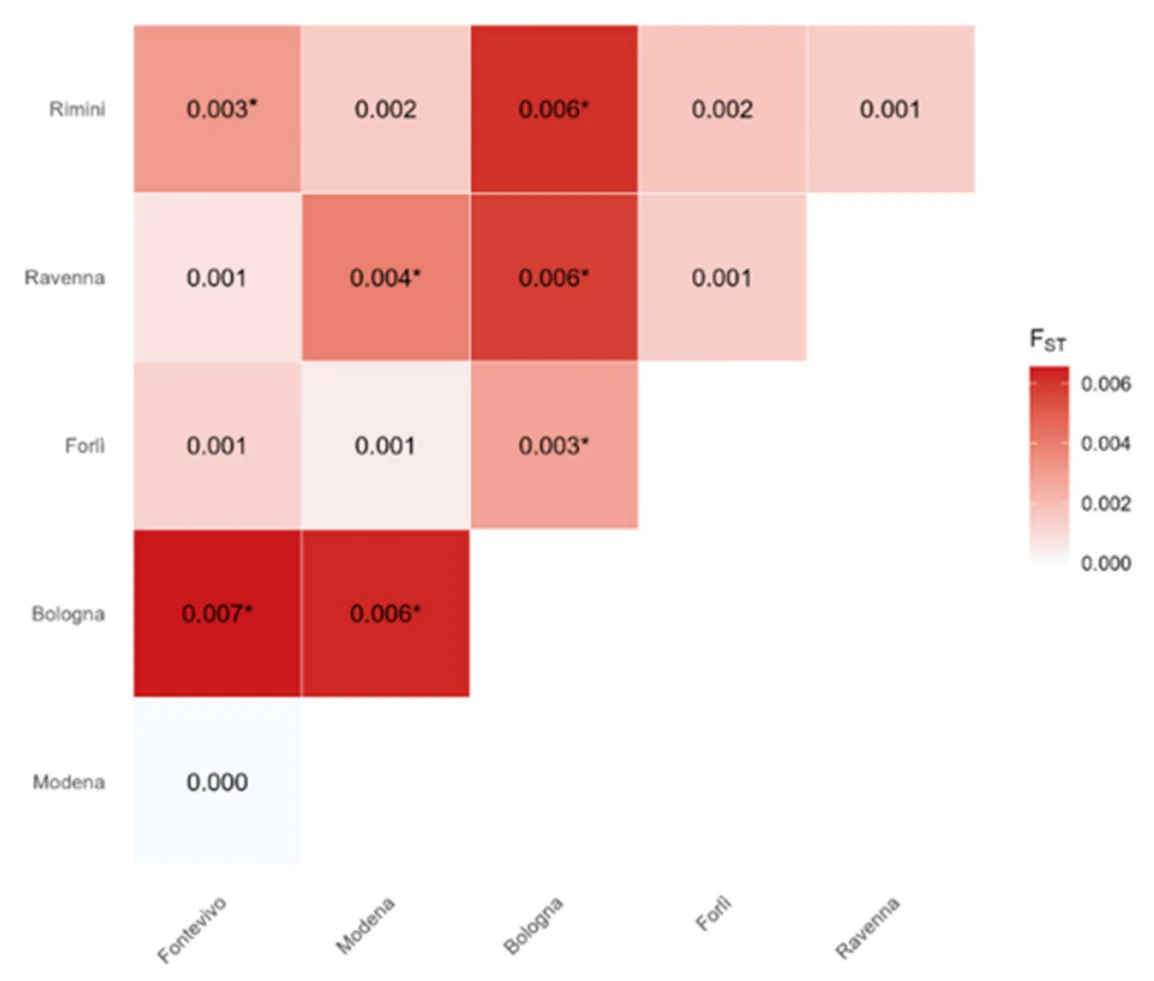
Pairwise F_ST_ matrix between the populations from the localities included in this study. On the right, the scale based on the lower (0.000) and higher (0.007) F_ST_ values calculated. * values are significant at *p* < 0.05.

Genetic differentiation among the sampled populations was generally low, with pairwise F_ST_ values spanning from 0.000 to 0.007 (Figure 6). The highest levels of differentiation were observed in comparisons involving the Bologna population, which exhibited statistically significant divergence (*p* < 0.05) against all other localities (F_ST_ = 0.003–0.007). Other than Bologna, significant differentiation was restricted to only two pairwise comparisons, Rimini—Fontevivo (F_ST_ = 0.003) and Ravenna—Modena (F_ST_ = 0.004). In contrast, non-significant F_ST_ values were widespread across the remaining pairs, with the lowest overall divergence observed between Fontevivo and Modena (F_ST_ = 0.000).

**Figure 7:**
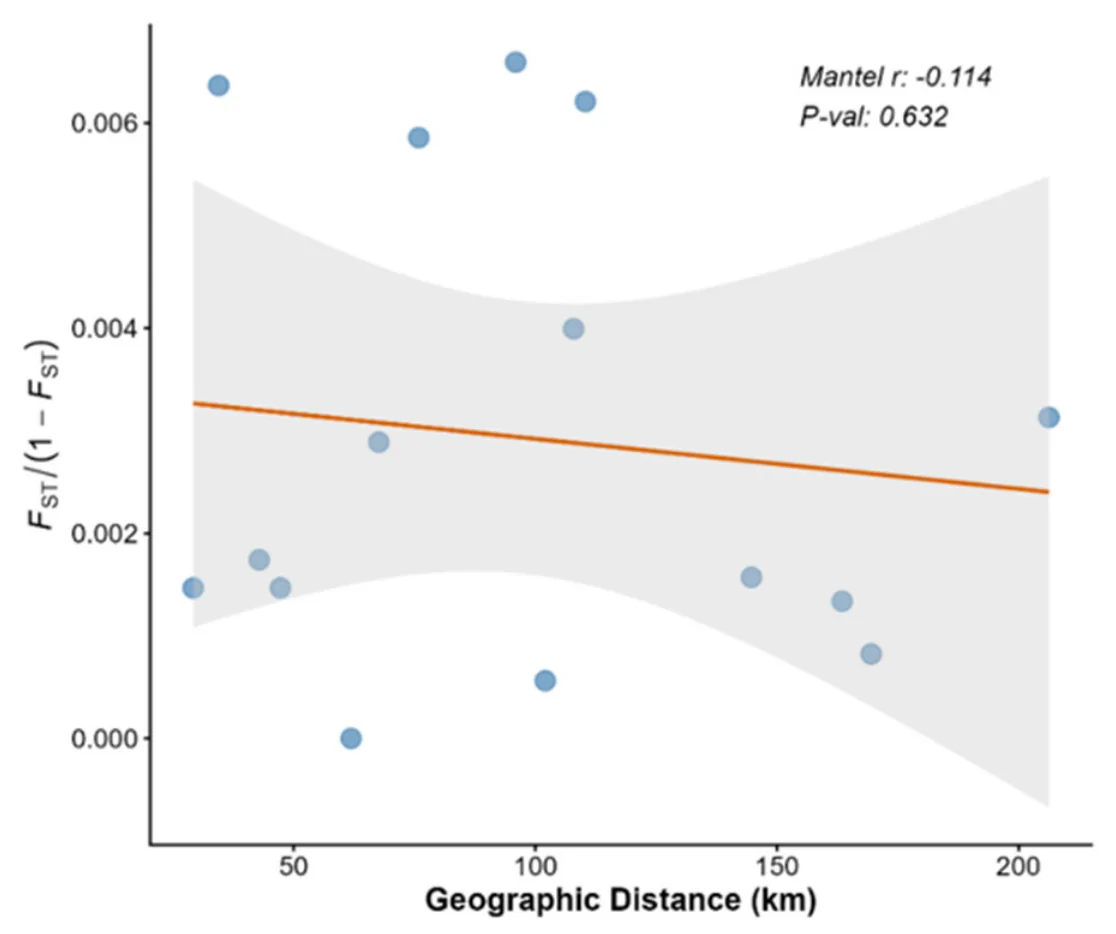
**Relationships between genetic and geographic distances.** Linearized pairwise differentiation [F_ST_/(1 − F_ST_)] as a function of geographic distance (km) among *Cx. pipiens* sampling sites. The red line and gray band represent the linear regression trend. Correlation parameters from the Mantel test are reported within the plot area (*p* > 0.05).

Pairwise genetic distances, linearized as F_ST_/(1 − F_ST_), were regressed against geographic distances across a spatial scale of approximately 210 km, spanning from Fontevivo to Rimini. The Mantel test revealed a weak, non-significant negative correlation between the genetic and geographic distance matrices (Mantel r = 0.114, *p* = 0.632; Figure 7). The lack of a positive linear relationship indicated that isolation by distance does not account for the observed genetic pattern.

### 3.4. DFB Resistance and Genomic Differentiation

The BLAST alignment of the Sanger sequence of the chs-1 gene fragment with the *Cx. pipiens* reference genome used in this study allowed us to identify the location of the locus, which lies approximately between 49 and 50 Mb on chromosome 3 (OZ004313.1).

**Figure 8:**
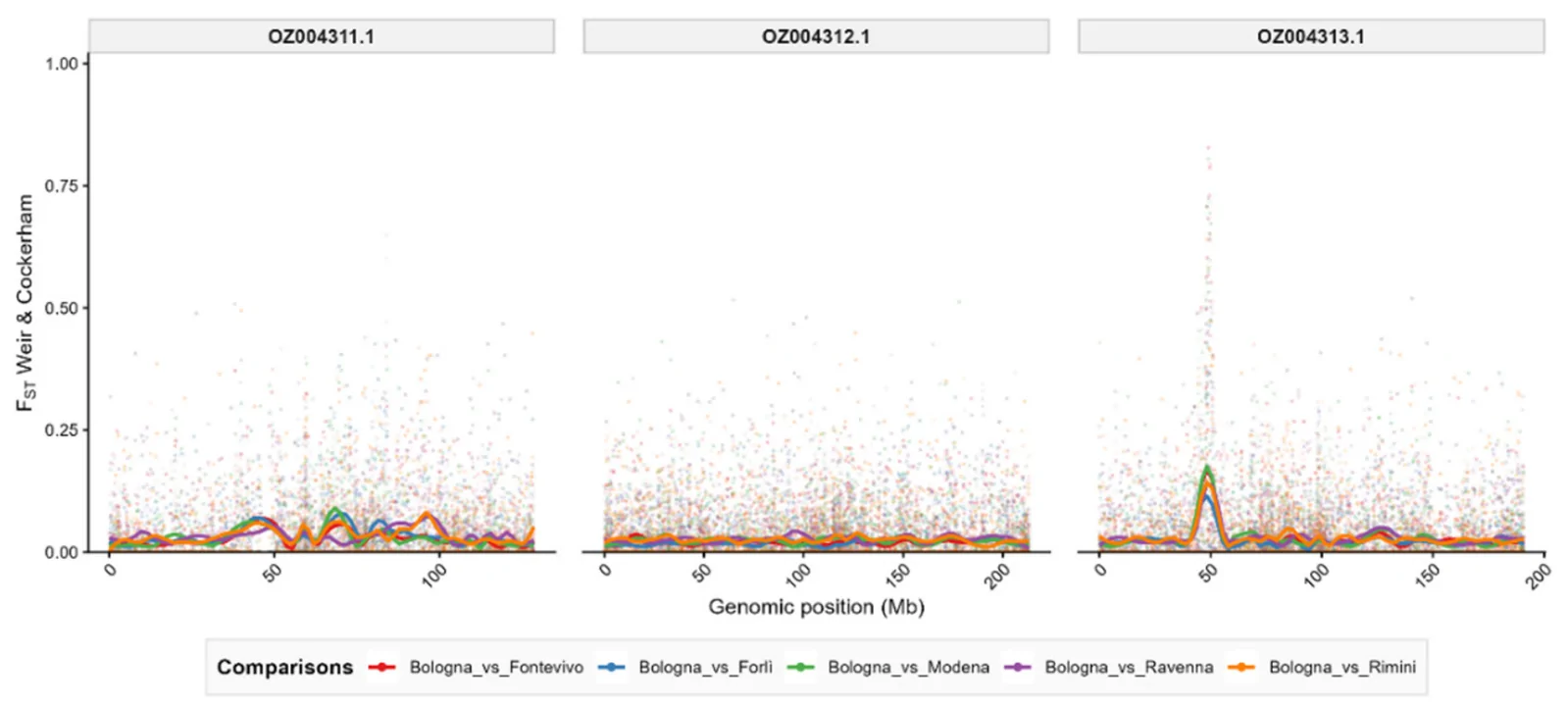
**Chromosomal-scale genetic differentiation (**$F_{ST}$**) profiles.** Windowed $F_{ST}$ profiles between Bologna and the five remaining populations across the three chromosomes OZ004311.1, OZ004312.1, and OZ004313.1. The *y*-axis represents Weir and Cockerham $F_{ST}$ values; the *x*-axis indicates genomic position in megabases (Mb). Dots represent individual 20 kb sliding windows, and solid lines show LOESS regression curves.

In the window-based genome scan, baseline $F_{ST}$ values across chromosomes OZ004311.1 and OZ004312.1 remained generally low (<0.15). Conversely, a localized $F_{ST}$ peak was detected on chromosome OZ004313.1 at approximately 50 Mb, overlapping with the mapped position of the chs-1 locus (Figure 8). This peak was present across all five pairwise comparisons involving the Bologna population (Bologna vs. Fontevivo, Forlì, Modena, Ravenna, and Rimini). Within this region, the smoothed LOESS regression curves reached maximum values between 0.15 and 0.20, whereas individual 20 kb genomic windows exhibited $F_{ST}$ values exceeding 0.80. The resulting figures of the other comparisons are provided in the Supplementary Materials (Figures S5–S9).

The multi-population analysis of the chs-1 locus yielded a global fixation index of $F_{ST}=0.4737$. Comparison against the ddRAD-seq reference dataset placed this value within the 99.98th percentile of the empirical background distribution (p=0.0002). At the population level, the localized allele frequency contrast (Δp) identified three locations as significant outliers exceeding the 95th percentile threshold (Table S3). The Bologna population exhibited the highest frequency of the resistance allele (q=0.8824), resulting in a localized contrast of Δ*p* = 0.7332, which fell within the 99.99th genomic percentile (*p* = 0.0001). Conversely, significant outlier signatures with lower-than-average allele frequencies were detected in Fontevivo (*q* = 0.0000, Δ*p* = 0.3983, 99.76th percentile, *p* = 0.0024) and Modena (*q* = 0.1250, Δ*p* = 0.2299, 97.25th percentile, *p* = 0.0275). The remaining three populations displayed intermediate frequencies of the resistance variant, ranging from 22.22% to 25.00%. The corresponding local contrasts (Δ*p* between 0.0758 and 0.1088) fell within the neutral genomic background, clustering between the 54.21st and 78.35th empirical percentiles (*p* > 0.05).

## 4. Discussion

In this study, we provided an updated analysis of the distribution and frequency of DFB-resistance-associated alleles in *Culex pipiens* across the Emilia-Romagna region (Italy) and assessed the population genetic structure of the species in this area.

Regarding the first issue, the screening of *Cx. pipiens* populations for chs-1-resistant mutations showed that DFB-resistance-associated alleles still occur in Emilia-Romagna and maintain a focal geographic distribution. Indeed, we identified all three target-site mutations known to confer DFB resistance within the region. At least one resistant mutation was identified in more than half the localities analyzed, and in four of them, multiple resistant mutations were detected (Figure 2; Table S1). Among the three DFB mutations, I1043L and I1043M showed the widest distribution, having been found from Modena to Rimini; conversely, I1043F was restricted to one population (i.e., Bologna). Notably, I1043M also showed the highest allele frequency in the region, exceeding 80% in Bologna. Although we cannot rule out the possibility that very-low-frequency mutations could be missed by our sampling, our results revealed a regional pattern concordant with that previously observed in Emilia-Romagna, showing higher mutation frequencies in the central-eastern than in the western provinces [14,18]. Interestingly, studies focusing outside Emilia-Romagna revealed that this pattern extends to other Italian regions. For example, Micocci et al. [13] found similar frequency ranges for DFB mutations in the Veneto region and locations that were almost entirely susceptible, co-occurring with a few locations showing higher frequencies of chs1-resistant alleles. Furthermore, as in our study, they found a geographic distribution of DFB-resistance-associated alleles characterized by a higher frequency of chs1-resistant alleles in coastal populations than in inland populations [13].

Regarding population structure, our genomic data showed a highly homogeneous gene pool at regional level. Indeed, we found low *F_ST_* values among the *Cx. pipiens* populations (*F_ST_* range = 0.000–0.007). Concordantly, DAPC and ADMIXTURE identified a single group (K = 1) as the optimal clustering solution, pointing toward a general absence of population structure. This general baseline of panmixia is also supported by internal genetic diversity metrics, such as a low and uniform genome-wide nucleotide diversity (π) across all sites. Furthermore, the *F_IS_* values were close to zero across all the populations, corroborating a scenario of genetic stability in the region, further confirmed by the HWE test. When genetic and geographic distance were correlated, a weak, non-significant negative correlation was observed, suggesting that genetic differentiation among populations is not shaped by geographic distance. A similar pattern has been observed in other mosquito species occurring in the same area [53]. Yet, it is concordant with what was previously observed in *Cx. pipiens*, where several hundred kilometers (>500 km) are required to generate isolation by distance [54,55], a condition that is not satisfied in our study area, as the maximum distance between sampling sites never exceeds 250 km. The landscape features, therefore, do not represent a limitation for gene flow in the Emilia-Romagna region.

This overall panmictic population structure contrasted with the spatial distribution of resistance alleles, which instead exhibited a distinct geographic pattern. Both outlier analyses identified chs-1 as a likely locus under local selection, indicating that the geographic distribution of resistance alleles is driven by localized selective pressures rather than by the underlying population structure. Accordingly, resistance alleles reached their highest frequency in the Bologna population and their lowest in the Modena and Fontevivo populations. Consistent with our findings, previous studies suggested that the pattern of DFB-resistance-associated alleles across the Emilia-Romagna region results from different selective pressures between the central-eastern and western provinces [18,56]. In recent years, DFB has been largely used in the Emilia-Romagna region for agricultural pest control [18,56]. In the central-eastern provinces, the higher orchard extension than in western provinces likely led to an increased DFB exposure of *Cx. pipiens* populations, resulting in a higher selective pressure on the chs-1 mutated alleles. Then, after the introduction of DFB in mosquito control applications, this initial selection was further boosted, leading to the observed pattern of DFB-resistance-associated alleles [18,56]. However, since the chs-1 mutation alone is likely insufficient to fully account for the slight overall genomic differentiation observed in Bologna, and because the width of the peak in chromosome 3 involved not only the chs-1 gene, it is highly probable that the resistant phenotype involves a more complex genetic architecture (including additional co-selected mechanisms), which our ddRAD dataset, due to its intrinsic resolution limits, could not fully evaluate. Future studies will be necessary to fully map the genomic extent of this local adaptation and clarify its direct relationship with localized selective pressures.

Taken together, our results have important implications for the incidence and management of insecticide resistance in *Cx. pipiens*. By revealing that DFB-resistant alleles are still present in Emilia-Romagna and considering a scenario of panmixia that poses the risk of their spreading across the region, our findings stress the importance of periodic monitoring in the long term. Another relevant issue to consider in better understanding the processes underlying DFB resistance is fitness cost. Fitness costs associated with insecticide resistance have been widely reported in arthropods, including IGR-resistant species [57,58,59,60]. Recently, reduced winter larval survival rates and increased larval developmental times have been found in *Cx. pipiens* mosquitoes artificially selected for three generations of DFB [61]. However, many factors outside laboratory conditions may affect overall fitness [62,63]. Field DFB-resistant mosquitoes are characterized by a complex phenotype involving unique molecular and phenotypic features [12]. Indeed, along with target-site mutations, metabolic differences and cuticular modifications have been detected [12,64]. Future studies investigating wild populations of *Cx. pipiens* will need therefore to fully understand the fitness cost associated with DFB resistance. Notably, DFB was withdrawn for mosquito control in Italy after June 2025 [13]. Because selective pressure has been removed, a decline in DFB-resistant alleles and reversion to susceptibility in field populations are expected in the coming years if resistant alleles carry a fitness cost [60]. Integrating resistance monitoring into fitness studies will be insightful for understanding this issue and assessing whether factors other than insecticide pressure may have contributed to the maintenance and spread of resistance.

## Figures and Tables

**Table 1:** **Sampling of *Culex pipiens* across the Emilia-Romagna region**. The localities and geographical coordinates where mosquito samples were collected are shown. The samples analyzed by ddRAD were sampled in June 2023 and September 2024; DFB-resistance screening includes all the samples analyzed by ddRAD and further samples collected in July 2024.

Locality	N	Year/Month	Long.	Lat.	ddRAD/Chs-1 Allele	
1. Piacenza (PC)	24	2024 July	9.662862	45.033134	-	chs-1 allele
2. Fontevivo (PR)	15	2023 June	10.1758527	44.8543083	ddRAD	chs-1 allele
3. Parma (PR)	24	2024 July	10.353992	44.789169	-	chs-1 allele
4. Reggio-Emilia (RE)	26	2024 July	10.608606	44.74115	-	chs-1 allele
5. Modena (MO)	30	2024 July	10.912015	44.665433	-	chs-1 allele
	12	2024 September			ddRAD	chs-1 allele
6. Bologna (BO)	7	2023 June	11.2833519	44.5043642	ddRAD	chs-1 allele
	28	2024 July			-	chs-1 allele
	10	2024 September			ddRAD	chs-1 allele
7. Ravenna (RA)	36	2024 July	12.233609	44.436349		chs-1 allele
	8	2024 September			ddRAD	chs-1 allele
8. Forlì (FR)	34	2024 July	12.032135	44.217346	-	chs-1 allele
	13	2024 September			ddRAD	chs-1 allele
9. Rimini (RI)	31	2024 July	12.526594	44.067529	-	chs-1 allele
	9	2024 September			ddRAD	chs-1 allele

## Data Availability

The sequencing reads generated in this study are deposited in the European Nucleotide Archive (ENA) under the accession id PRJEB115311.

## Supplementary Materials

The following supporting information can be downloaded at: https://www.mdpi.com/article/10.3390/insects17080877/s1, Table S1: Genotype and allele frequency of chs-1 alleles associated with DFB resistance in *Culex pipiens* populations from the Emilia-Romagna region. Table S2: Genotype and allele frequency of chs-1 alleles associated with DFB resistance in *Culex pipiens* samples collected across the Emilia-Romagna region for ddRAD analyses. Table S3: Summary of empirical outlier analysis for the *chs*-1 locus across the six populations included in the ddRAD dataset. Figure S1: ADMIXTURE bar plots for cluster solutions from K 1 to 8 on the filtered VCF (3556 SNPs). Horizontal bar plots illustrate ancestral membership coefficients for individual mosquitoes. The labels on the left indicate the stability of the Pong modal assignment, representing the proportion of identical runs across 20 replicates. Figure S2: Population clustering via Discriminant Analysis of Principal Components (DAPC) on the Stacks populations VCF (12,012 SNPs). The scatter plot illustrates the genetic relationships among individuals based on the first two discriminant functions (LD1 and LD2), which account for 60.3% and 21.88% of the total genetic variance, respectively. Dots represent individual genotypes, ellipses define the 95% confidence intervals for each population cluster, and colored diamonds represent group centroids. The color-legend for the populations analyzed is on the right. Figure S3: Cross-validation error calculated by the ADMIXTURE analysis on the Stacks populations VCF (12,012 SNPs) showing the best K value. Figure S4: ADMIXTURE bar plots for cluster solutions from K 1 to 8 on the Stacks populations VCF (12,012 SNPs). Horizontal bar plots illustrate ancestral membership coefficients for individual mosquitoes. The labels on the left indicate the stability of the Pong modal assignment, representing the proportion of identical runs across 20 replicates. Figure S5: Chromosomal-scale genetic differentiation (F_ST_) profiles—Fontevivo. Windowed F_ST_ profiles between Fontevivo and the five remaining populations across the three chromosomes OZ004311.1, OZ004312.1, and OZ004313.1. The *y*-axis represents Weir and Cockerham F_ST_ values; the *x*-axis indicates genomic position in megabases (Mb). Dots represent individual 20 kb sliding windows, and solid lines show LOESS regression curves. Figure S6: Chromosomal-scale genetic differentiation (F_ST_) profiles—Forlì. Windowed F_ST_ profiles between Forlì and the five remaining populations across the three chromosomes OZ004311.1, OZ004312.1, and OZ004313.1. The *y*-axis represents Weir and Cockerham F_ST_ values; the *x*-axis indicates genomic position in megabases (Mb). Dots represent individual 20 kb sliding windows, and solid lines show LOESS regression curves. Figure S7: Chromosomal-scale genetic differentiation (F_ST_) profiles—Modena. Windowed F_ST_ profiles between Modena and the five remaining populations across the three chromosomes OZ004311.1, OZ004312.1, and OZ004313.1. The *y*-axis represents Weir and Cockerham F_ST_ values; the *x*-axis indicates genomic position in megabases (Mb). Dots represent individual 20 kb sliding windows, and solid lines show LOESS regression curves. Figure S8: Chromosomal-scale genetic differentiation (F_ST_) profiles—Ravenna. Windowed F_ST_ profiles between Ravenna and the five remaining populations across the three chromosomes OZ004311.1, OZ004312.1, and OZ004313.1. The *y*-axis represents Weir and Cockerham F_ST_ values; the *x*-axis indicates genomic position in megabases (Mb). Dots represent individual 20 kb sliding windows, and solid lines show LOESS regression curves. Figure S9: Chromosomal-scale genetic differentiation (F_ST_) profiles—Rimini. Windowed F_ST_ profiles between Rimini and the five remaining populations across the three chromosomes OZ004311.1, OZ004312.1, and OZ004313.1. The *y*-axis represents Weir and Cockerham F_ST_ values; the *x*-axis indicates genomic position in megabases (Mb). Dots represent individual 20 kb sliding windows, and solid lines show LOESS regression curves.

## Abbreviations

The following abbreviations are used in this manuscript:

BPUs	Benzoylureas
CTAB	Cetyltrimethylammonium bromide
CV	Cross-validation
DAPC	Discriminant analysis of principal components
ddRAD	Double digest restriction-site associated DNA
DFB	Diflubenzuron
FDR	False discovery rate
HWE	Hardy–Weinberg equilibrium
IBD	Isolation by distance
IGRs	Insect growth regulators
LD	Linkage disequilibrium
SNPs	Single nucleotide polymorphisms
VCF	Variant call format
