# Supplementary material for: Allelic Survey and Population Genomics in a Hotspot of Diflubenzuron Resistance in the Mosquito Culex pipiens
Source: Insects. 2026 Aug 21;17(8):877. doi: 10.3390/insects17080877 (PMC13512999; doi:10.3390/insects17080877)
Supplement: Supplementary file 1 [file insects-17-00877-s001.zip › insects-4422774-supplementary.pdf]

Table S1. Genotype and allele frequency of chs-1 alleles associated with DFB resistance in *Culex pipiens* populations from the Emilia-Romagna region.

	**N**	**Genotipi**										**Frequenze alleliche**			
**Locality**		**II**	**IL**	**LL**	**IM**	**MM**	**LM**	**FF**	**IF**	**FL**	**FM**	**I**	**L**	**M**	**F**
Piacenza (PC)	24	24	-	-	-	-	-	-	-	-	-	1.00	-	-	-
Fontevivo (FO)	15	15										1.00			
Parma (PR)	24	24	-	-	-	-	-	-	-	-	-	1.00	-	-	-
Reggio-Emilia (RE)	26	26	-	-	-	-	-	-	-	-	-	1.00	-	-	-
Modena (MO)	42	30	2	2	-	-	-	-	-	-	-	0.93	0.07	-	-
Bologna (BO)	45	1	-	-	4	31	1	-	-	-	8	0.07	0.01	0.84	0.08
Ravenna (RA)	44	35	4	-	5	-	-	-	-	-	-	0.90	0.04	0.06	-
Forlì (FR)	47	34	5	-	6	1	1	-	-	-	-	0.84	0.09	0.07	-
Rimini (RI)	40	22	5	-	11	-	2	-	-	-	-	0.75	0.09	0.16	-

Table S2. Genotype and allele frequency of chs-1 alleles associated with DFB resistance in *Culex pipiens* samples collected across the Emilia-Romagna region for ddRAD analyses.

	**N**	**Genotipi**										**Frequenze alleliche**			
**Località**		**II**	**IL**	**LL**	**IM**	**MM**	**LM**	**FF**	**IF**	**FL**	**FM**	**I**	**L**	**M**	**F**
Fontevivo (FO)	15	15	-	-	-	-	-	-	-	-	-	1.00	-	-	-
Modena (MO)	12	10	1	1	-	-	-	-	-	-	-	0.87	0.12	-	-
Bologna (BO)	17	1	-	-	2	10	1	-	-	-	3	0.09	0.03	0.76	0.09
Ravenna (RA)	8	4	2	-	2	-	-	-	-	-	-	0.75	0,12	0,12	-
Forlì (FR)	13	8	2	-	2	1	-	-	-	-	-	0.77	0.08	0.15	-
Rimini (RI)	9	5	2	-	2	-	-	-	-	-	-	0.77	0,11	0,11	-

Table S3. Summary of the empirical outliers analysis for the *chs*-1 locus across the six populations included in the ddRAD dataset.

Location	Samples (N)	chs-1 Resist Freq. (q)	Δp chs-1	Empirical Percentile (%)	p-value	Assigned Status
**Bologna**	17	0.8824	0.7332	99.99	0.0001	Strong outliers ($p < 0.01$)
**Fontevivo**	15	0.0000	0.3983	99.76	0.0024	Strong outliers ($p < 0.01$)
**Modena**	12	0.1250	0.2299	97.25	0.0275	Outliers ($p > 0.05$)
**Forlì**	13	0.2310	0.1050	78.35	0.2165	Neutral
**Rimini**	9	0.2222	0.1088	73.48	0.2652	Neutral
**Ravenna**	8	0.2500	0.0758	54.21	> 0.05	Neutral

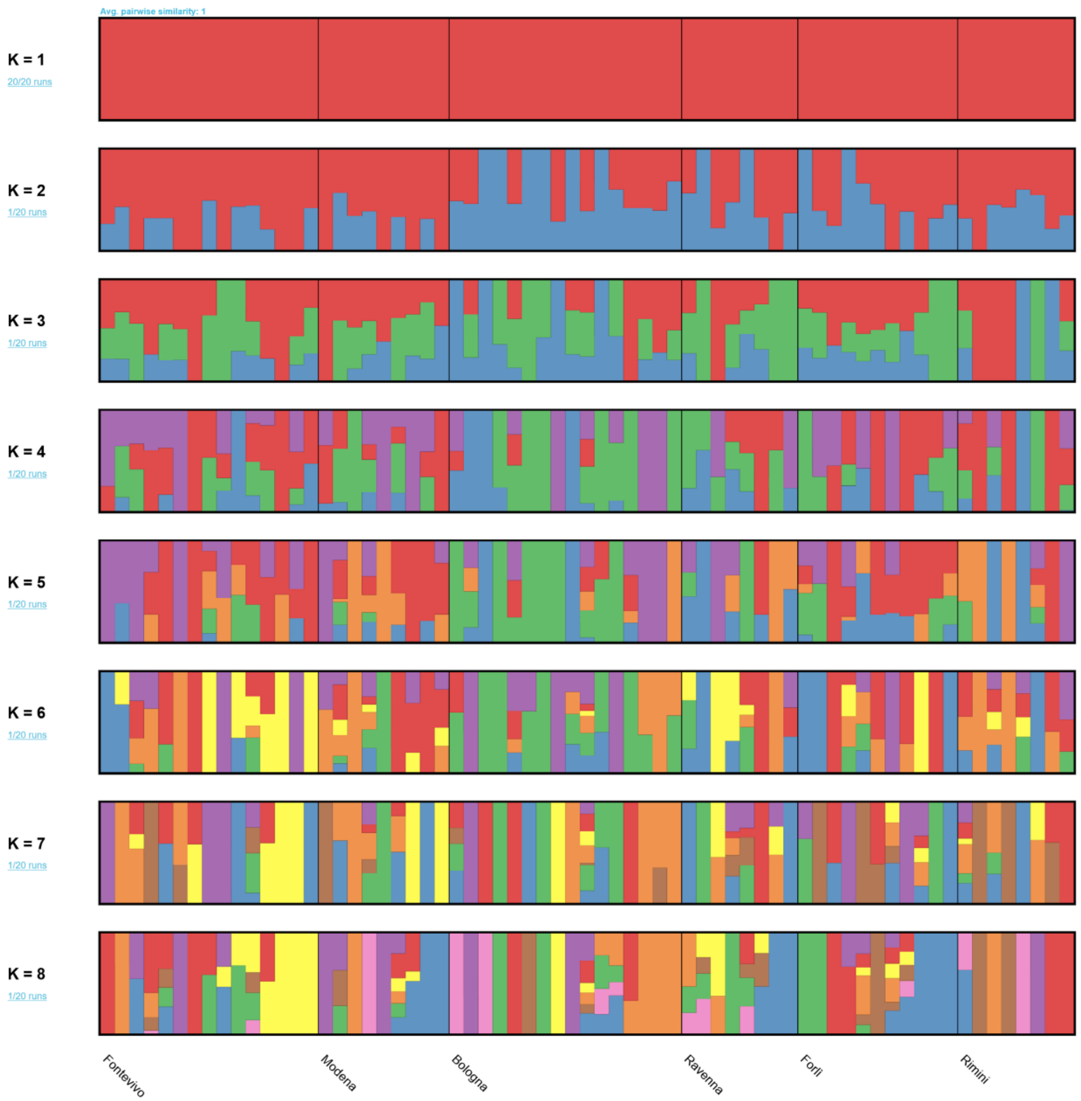

Figure S1. ADMIXTURE barplots for cluster solutions from K 1 to 8 on the filtered VCF (3,556 SNPs). Horizontal bar plots illustrate ancestral membership coefficients for individual mosquitoes. The labels on the left indicate the stability of the PONG modal assignment, representing the proportion of identical runs across 20 replicates.

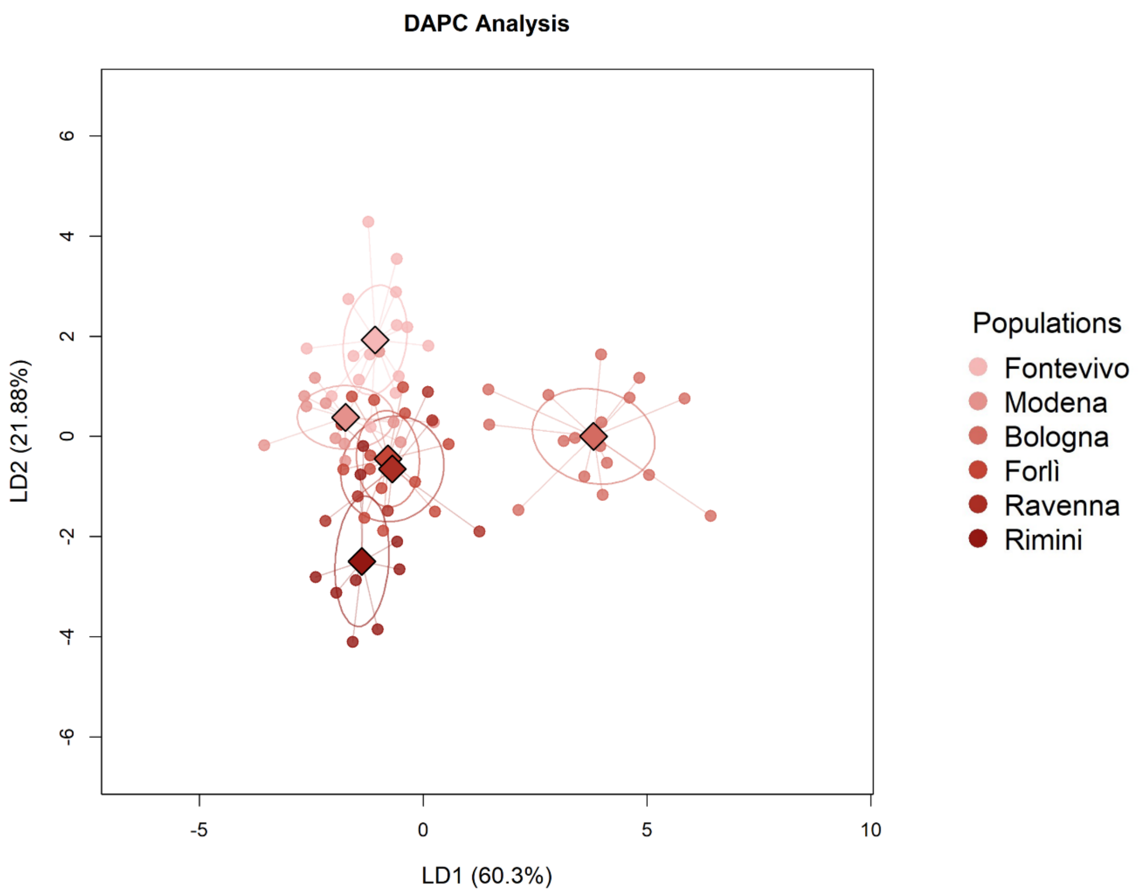

Figure S2. Population clustering via Discriminant Analysis of Principal Components (DAPC) on the stacks populations VCF (12,012 SNPs). The scatter plot illustrates the genetic relationships among individuals based on the first two discriminant functions (LD1 and LD2), which account for 60.3% and 21.88% of the total genetic variance, respectively. Dots represent in-dividual genotype, ellipses define the 95% confidence intervals for each popula-tion cluster, and coloured diamonds represent group centroids. On the right, the colour-legend for the populations analysed.

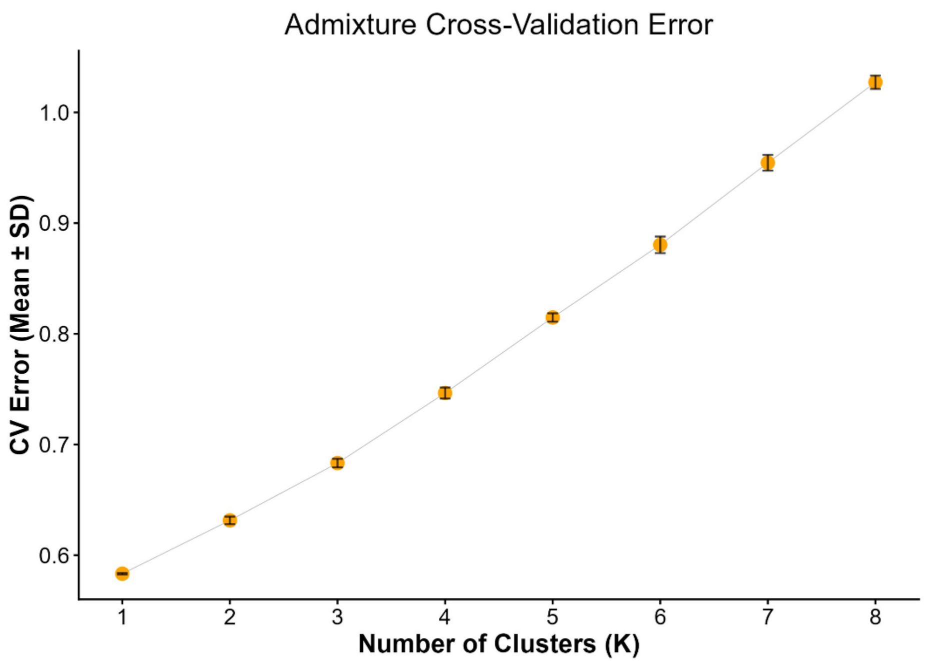

Figure S3. Cross-Validation error calculated by the ADMIXTURE analysis on the stacks populations VCF (12,012 SNPs) showing the best K value.

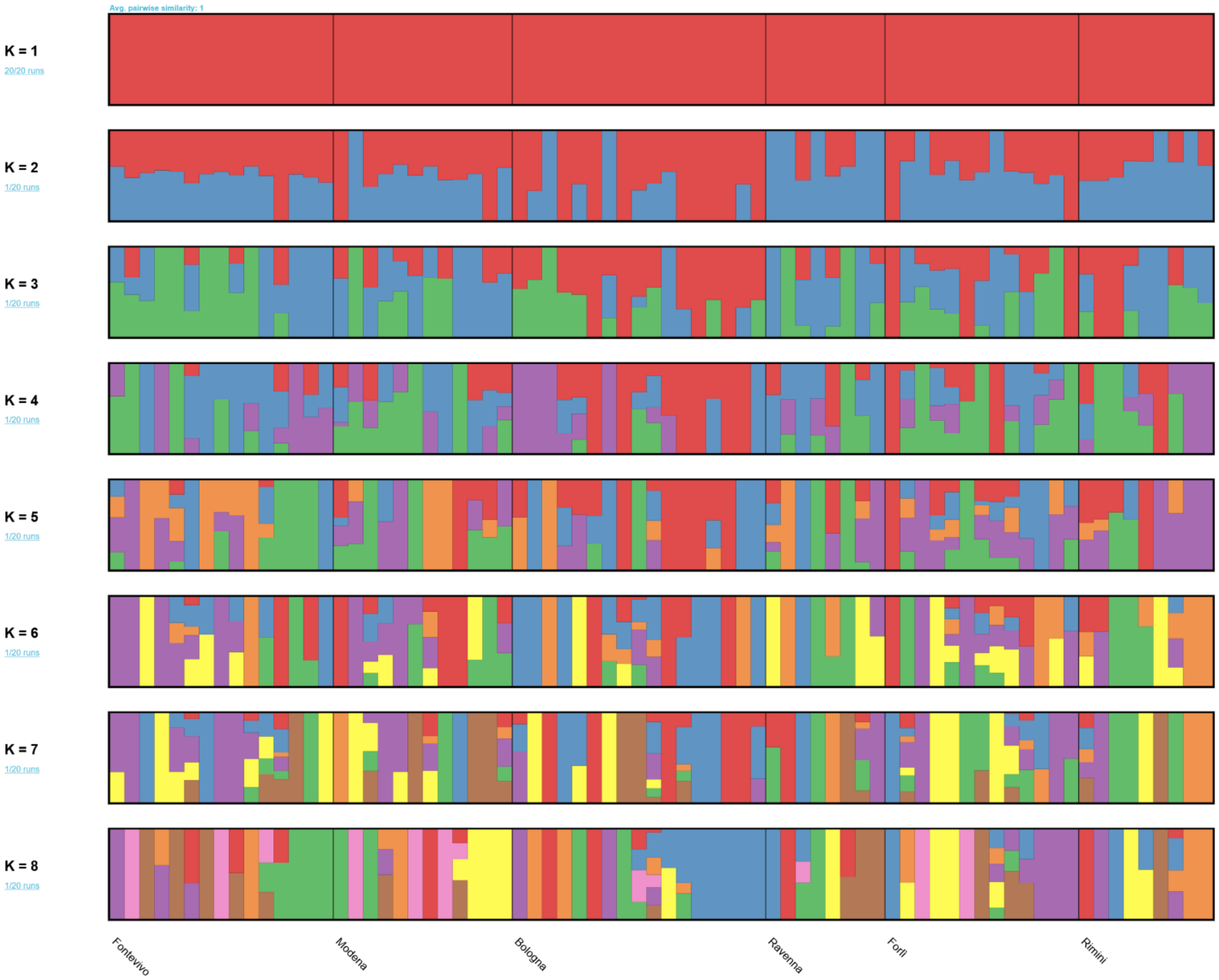

Figure S4. ADMIXTURE barplots for cluster solutions from K 1 to 8 on the stacks populations VCF (12,012 SNPs). Horizontal bar plots illustrate ancestral membership coefficients for individual mosquitoes. The labels on the left indicate the stability of the PONG modal assignment, representing the proportion of identical runs across 20 replicates.

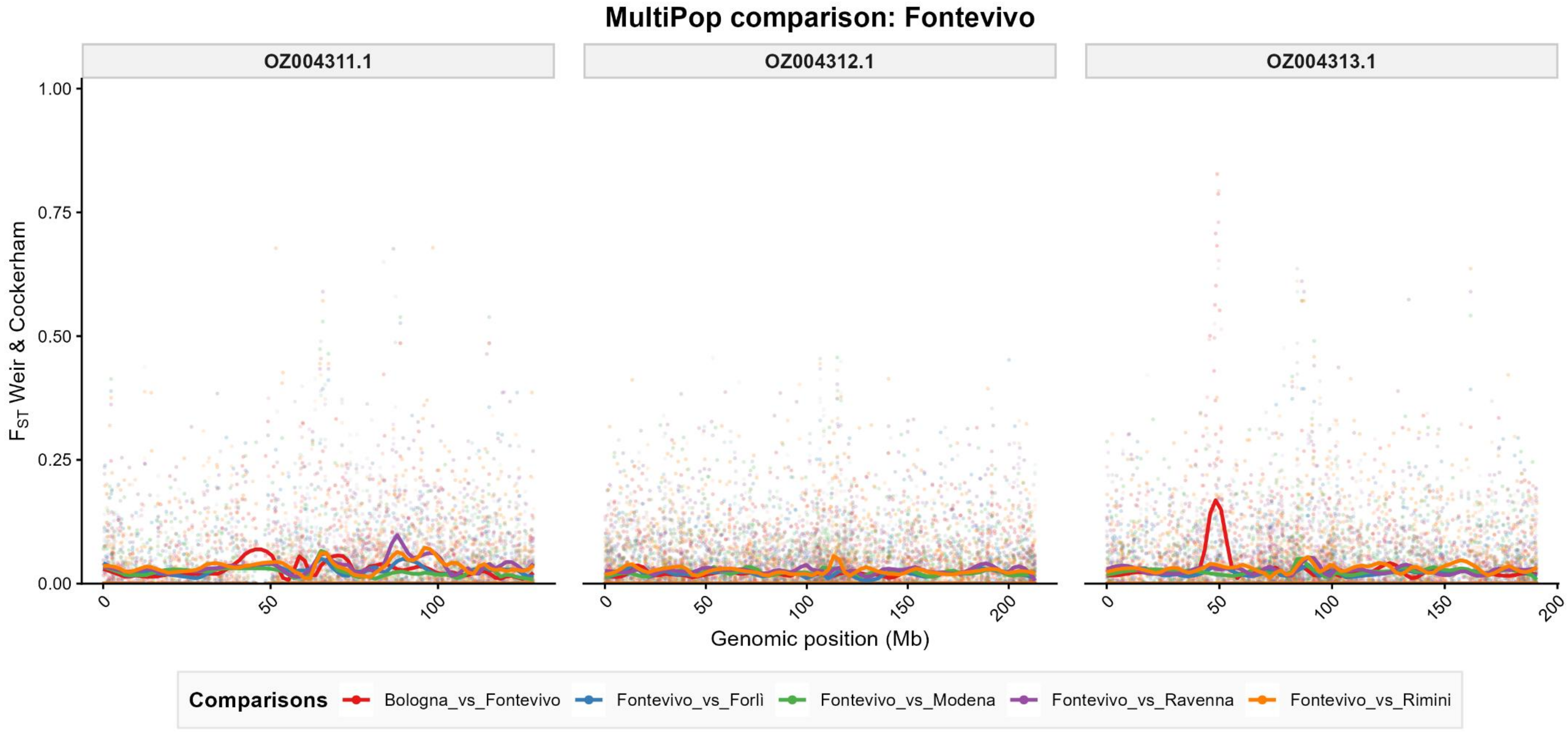

Figure S5. **Chromosomal-scale genetic differentiation (F_{ST}) profiles.** Windowed F_{ST} profiles between Fontevivo and the five remaining populations across the 3 chromosomes OZ004311.1, OZ004312.1, and OZ004313.1. The y-axis represents Weir and Cockerham F_{ST} values; the x-axis indicates genomic position in Megabases (Mb). Dots represent individual 20-kb sliding windows and solid lines show LOESS regression curves.

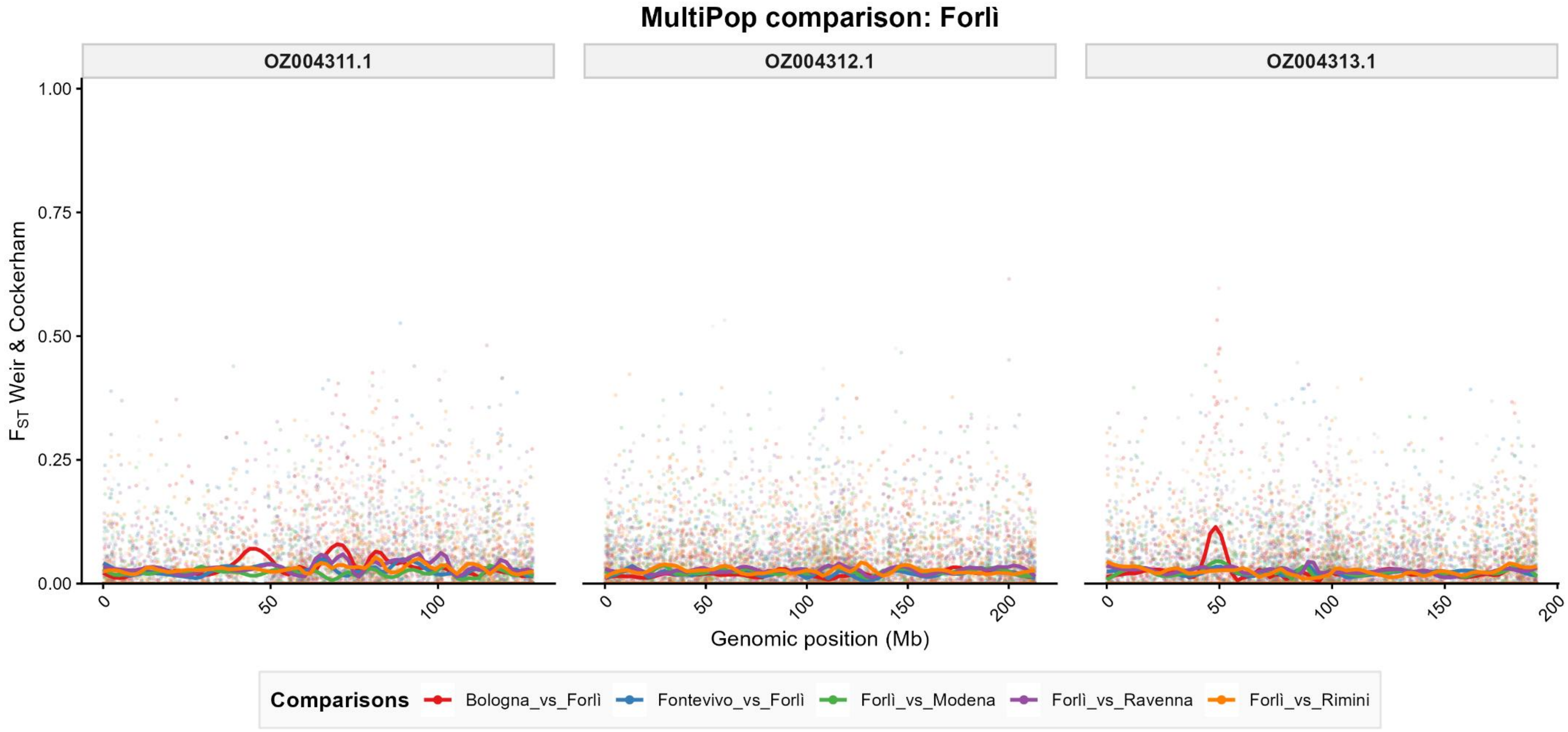

Figure S6. **Chromosomal-scale genetic differentiation (F_{ST}) profiles.** Windowed F_{ST} profiles between Forlì and the five remaining populations across the 3 chromosomes OZ004311.1, OZ004312.1, and OZ004313.1. The y-axis represents Weir and Cockerham F_{ST} values; the x-axis indicates genomic position in Megabases (Mb). Dots represent individual 20-kb sliding windows and solid lines show LOESS regression curves.

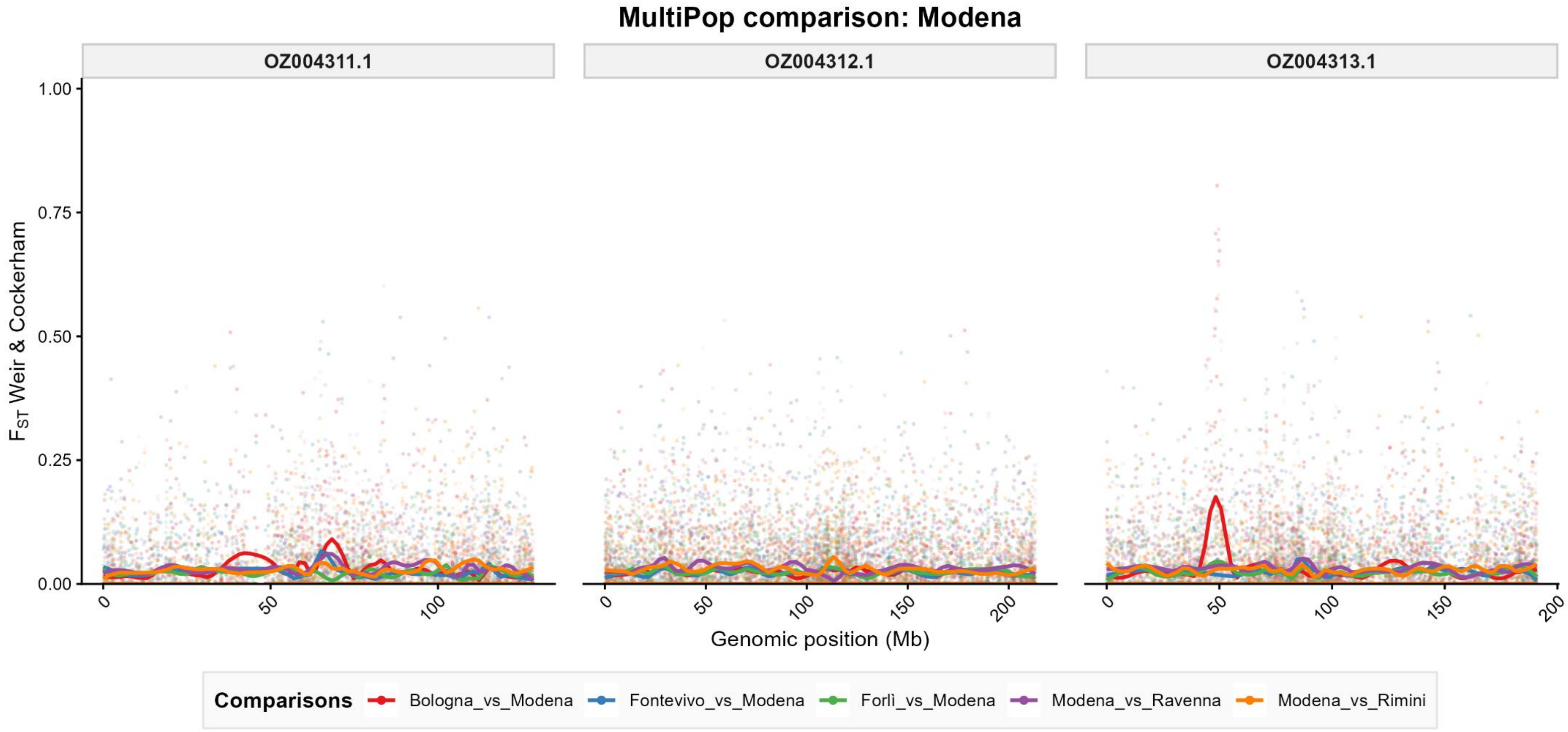

Figure S7. **Chromosomal-scale genetic differentiation (F_{ST}) profiles.** Windowed F_{ST} profiles between Modena and the five remaining populations across the 3 chromosomes OZ004311.1, OZ004312.1, and OZ004313.1. The y-axis represents Weir and Cockerham F_{ST} values; the x-axis indicates genomic position in Megabases (Mb). Dots represent individual 20-kb sliding windows and solid lines show LOESS regression curves.

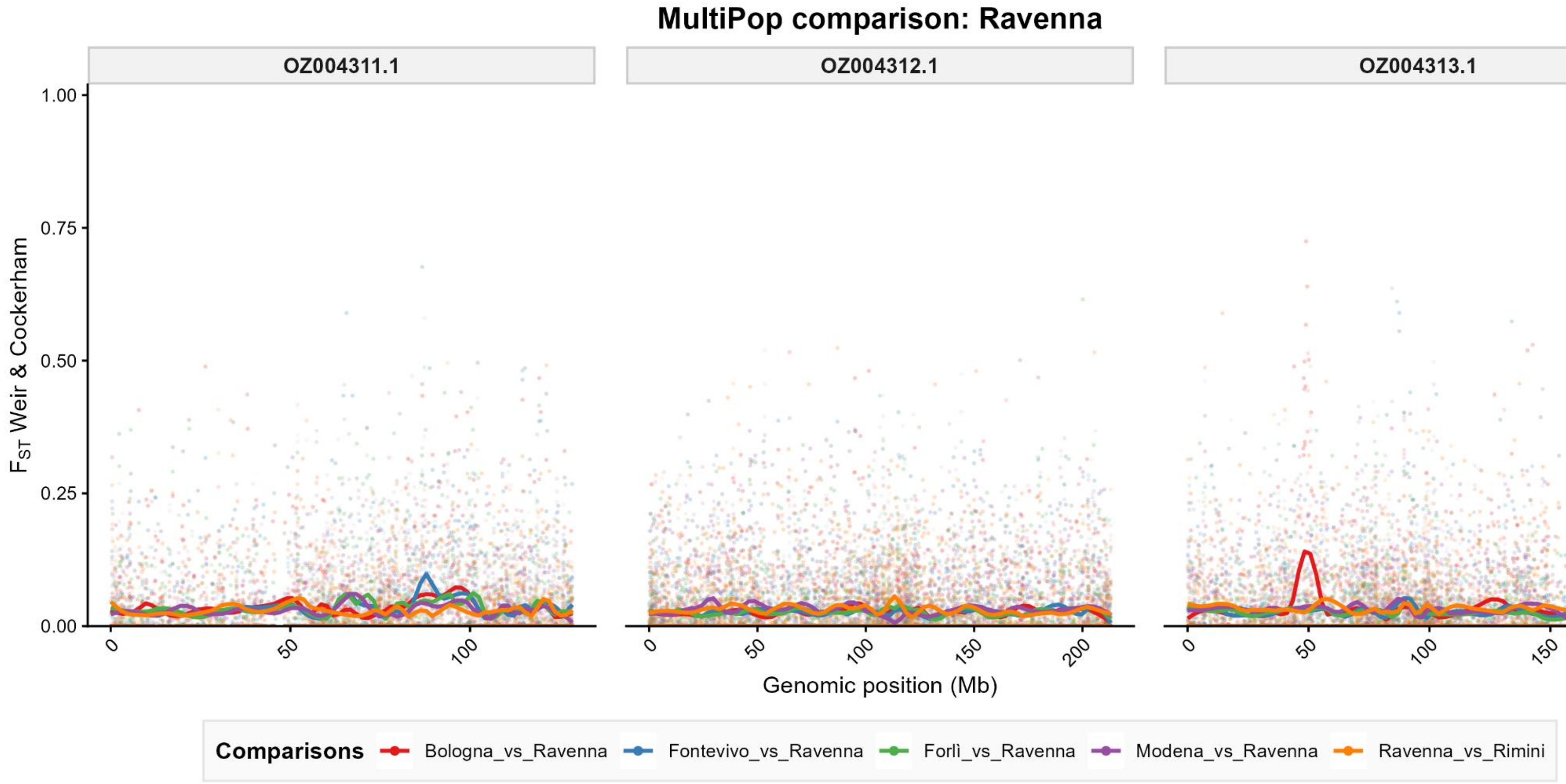

Figure S8. **Chromosomal-scale genetic differentiation (F_{ST}) profiles.** Windowed F_{ST} profiles between Ravenna and the five remaining populations across the 3 chromosomes OZ004311.1, OZ004312.1, and OZ004313.1. The y-axis represents Weir and Cockerham F_{ST} values; the x-axis indicates genomic position in Megabases (Mb). Dots represent individual 20-kb sliding windows and solid lines show LOESS regression curves.

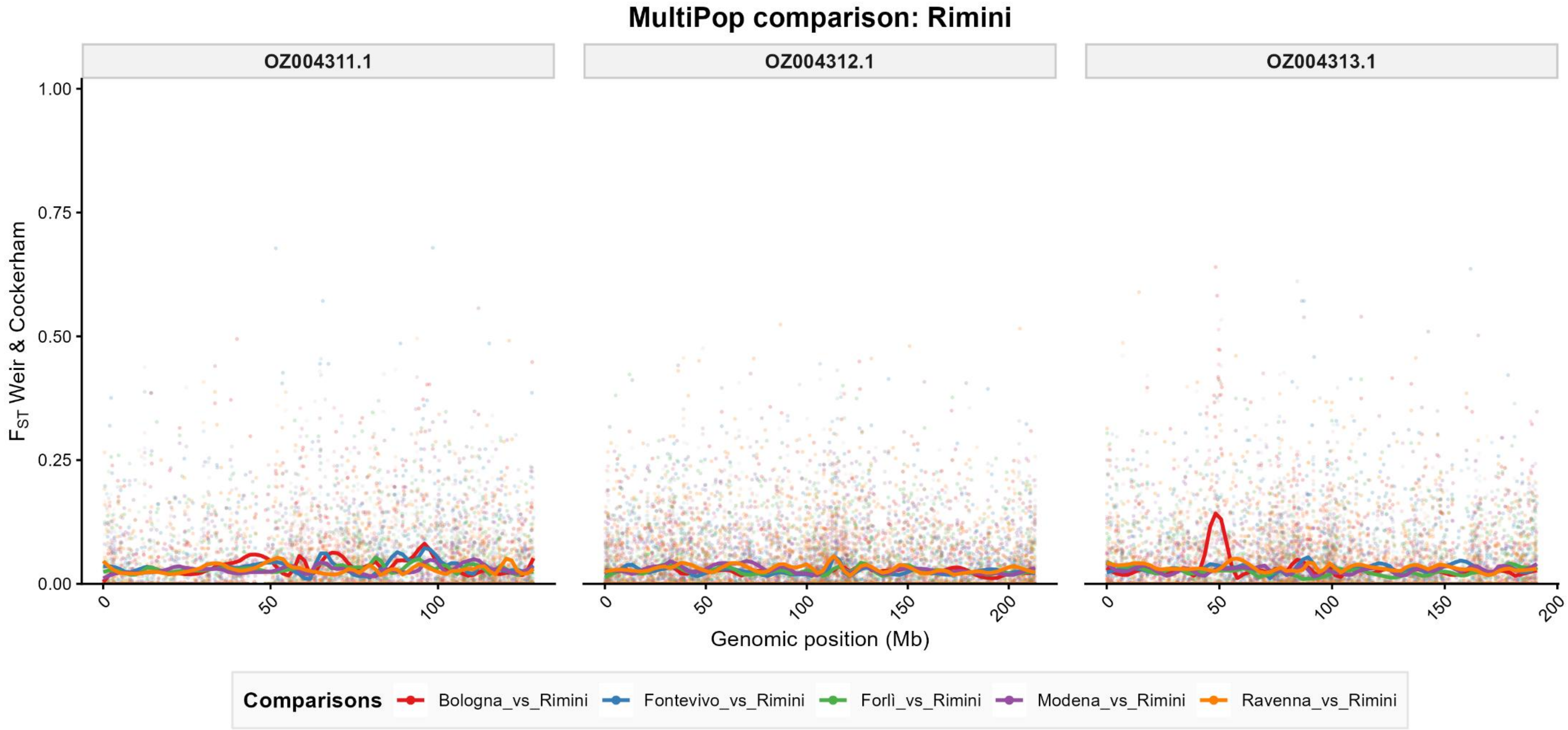

Figure S9. **Chromosomal-scale genetic differentiation (F_{ST}) profiles.** Windowed F_{ST} profiles between Rimini and the five remaining populations across the 3 chromosomes OZ004311.1, OZ004312.1, and OZ004313.1. The y-axis represents Weir and Cockerham F_{ST} values; the x-axis indicates genomic position in Megabases (Mb). Dots represent individual 20-kb sliding windows and solid lines show LOESS regression curves.
